# Effect of inhibition of the Ubiquitin-Proteasome System and Hsp90 on growth and survival of Rhabdomyosarcoma cells *in vitro*

**DOI:** 10.1186/1471-2407-12-233

**Published:** 2012-06-12

**Authors:** Marica Peron, Paolo Bonvini, Angelo Rosolen

**Affiliations:** 1 Clinica di Oncoematologia Pediatrica, Azienda Ospedaliera-Università di Padova, Via Giustiniani 3, Padova, 35128, Italy; 2 Fondazione Città della Speranza, Via del Lavoro 12, Malo-Vicenza, 36034, Italy

## Abstract

**Background:**

The ubiquitin-proteasome system (UPS) and the heat shock response (HSR) are two critical regulators of cell homeostasis, as their inhibition affects growth and survival of normal cells, as well as stress response and invasiveness of cancer cells. We evaluated the effects of the proteasome inhibitor Bortezomib and of 17-DMAG, a competitive inhibitor of Hsp90, in rhabdomyosarcoma (RMS) cells, and analyzed the efficacy of single-agent exposures with combination treatments.

**Methods:**

To assess cytotoxicity induced by Bortezomib and 17-DMAG in RMS cells, viability was measured by MTT assay after 24, 48 and 72 hours. Western blotting and immunofluorescence analyses were carried out to elucidate the mechanisms of action. Apoptosis was measured by FACS with Annexin-V-FITC and Propidium Iodide.

**Results:**

Bortezomib and 17-DMAG, when combined at single low-toxic concentrations, enhanced growth inhibition of RMS cells, with signs of autophagy that included intensive cytoplasmic vacuolization and conversion of cytosolic LC3-I protein to its autophagosome-associated form. Treatment with lysosomal inhibitor chloroquine facilitates apoptosis, whereas stimulation of autophagy by rapamycin prevents LC3-I conversion and cell death, suggesting that autophagy is a resistance mechanism in RMS cells exposed to proteotoxic drugs. However, combination treatment also causes caspase-dependent apoptosis, PARP cleavage and Annexin V staining, as simultaneous inhibition of both UPS and HSR systems limits cytoprotective autophagy, exacerbating stress resulting from accumulation of misfolded proteins.

**Conclusion:**

The combination of proteasome inhibitor Bortezomib with Hsp90 inhibitor 17-DMAG, appears to have important therapeutic advantages in the treatment of RMS cells compared with single-agent exposure, because compensatory survival mechanisms that occur as side effects of treatment may be prevented.

## Background

Rhabdomyosarcoma (RMS) is the most common sarcoma among children and adolescents, accounting for 5 % of all malignancies of these age groups. RMS can be distinguished in alveolar (ARMS), embryonal (ERMS), and the less common variant pleomorphic RMS subtypes. ARMS are more aggressive than ERMS, have a higher tendency to metastasize [1,2] and frequently localize in the extremities [3]. ERMS mainly originate in the genitourinary tract, head and neck [4] and have a better prognosis than ARMS. In 2/3 of cases ARMS cells harbour a reciprocal chromosomal translocation t (2;13)(q35;q14) [5] that generates the chimeric transcriptional factor PAX3-FKHR, which causes aberrant gene expression in RMS cells and influences tumour aggressiveness [6].

Recently, Bortezomib and 17-DMAG have been suggested as potential new agents for the treatment of RMS, being both drugs effective at reducing RMS cell survival and invasiveness [7,8]. Bortezomib (Velcade^TM^) is a dipeptidyl boronic acid derivative, that inhibits the chymotryptic-like activity of the 26S proteasome subunit, and promotes apoptosis through G_2_/M cell cycle arrest, activation of stress response and impairment of NF-κB signalling [9]. Bortezomib-dependent inhibition of proteasome activity is a therapeutic strategy under investigation in several tumour types, used either as single agent or in combination with conventional chemotherapeutic agents [10,11]. 17-DMAG [17-(Dimethylaminoethylamino)-17-Demethoxygeldanamycin] is a soluble geldanamycin derivative [12], a benzoquinoid ansamycin antibiotic inhibitor of the Hsp90 molecular chaperone, which prevents nucleotide binding and ATPase activity of Hsp90 [13], thus impeding the correct folding of several signal transduction proteins involved in tumour cell growth and survival [14]. 17-DMAG has been studied for its antitumor activity in blastomas [15], carcinomas and leukemias [16], where it caused inhibition of cell growth and survival.

We used Bortezomib and 17-DMAG as single agents or in combination and we demonstrated that when added simultaneously they induce growth inhibition and cell death in rhabdomyosarcoma cells.

## Methods

### Cell cultures

Human RMS cell lines RD, RH30 were maintained in RPMI 1640 medium containing 10 % heat-inactivated fetal calf serum (FCS), 2 mmol/L glutamine, 100 U/mL penicillin and 100 μg/mL streptomycin and grown under standard tissue-culture conditions.

### Reagents and antibodies

17-DMAG was purchased from Alexis (Axxora Life Science, USA), dissolved in dimethylsulfoxide (DMSO) at concentration of 10 mM and stored at −80 °C.

Bortezomib was kindly provided by Millenium Pharmaceuticals (Millenium Pharmaceuticals, Inc. Cambridge, Massachusetts, USA). Antibodies against PARP and LC3B were purchased from Cell Signaling (Cell Signaling Technology, Inc., Danvers, Massachusetts, USA), β-actin, PMSF, chloroquine and rapamycin from SIGMA (SIGMA-Aldrich Co., St. Louis, Missouri, USA). Leupeptin and aprotinin protease inhibitors were obtained from CAPPEL (ICN Biomedicals Inc., Irvine, California, USA) and Calbiochem (Merck, Darmstadt, Germany), respectively. Acridine orange was purchased from Invitrogen (Invitrogen, Eugene, Oregon, USA), whereas DAPI nucleic acid stain, anti-tubulin antibody and fluorophore-conjugated goat anti-rabbit Alexa488 antibodies were from Molecular Probes (Invitrogen, Eugene, Oregon, USA). Horseradish peroxidase-conjugated sheep anti-mouse and donkey anti-rabbit antibodies were obtained from GE Healthcare (GE Healthcare Bio-Sciences AB, Uppsala, Sweden). For western blot analysis, proteins were quantified by the BCA protein assay (Pierce Chemical, Co., Rockford, Illinois, USA), transferred to nitrocellulose membranes (Schleicher & Schuell-Whatman, Maidstone, Kent, UK), and visualized by using Chemicon chemiluminescence reagents (Chemicon International, Inc., Temecula, California, USA).

### Cell viability assays

Cell viability was assessed by MTT assay. Briefly, 3x10^3^ cells were seeded in 96-well plates and cultured in the presence or absence of the test-drugs at 37 °C for up to 72 h. MTT salt ((3-(4,5-dimethylthiazol-2-yl)-2,5-diphenyltetrazolium bromide); SIGMA Co., USA) was added for 4 h and reduction of MTT salt was measured at 24 h intervals by spectrophotometer at 540 nm wave-length.

To determine apoptosis, cells were treated with drugs or left untreated as indicated. After 48h-treatment, 0,5x10^6^ cells were harvested and washed with temperate PBS. The cells were resuspended in 1 mL of 1X annexin-binding buffer (10 mM HEPES, pH 7.4; 140 mM NaOH; 2.5 mM CaCl_2_), stained with 5 μl annexin-V-fluorescein isothiocyanate (FITC) and 5 μl of propidium iodide (PI), and then incubated for 15 minutes at room temperature in the dark (Immunostep Research, Salamanca, Spain). Apoptotic cells were then determined by flow cytometry, using a FACS Calibur (Becton Dickinson, Franklin Lakes, New Jersey, USA). Both early apoptotic (annexin-V-positive, PI-negative) and late apoptotic cells (annexin-V-positive, PI-positive) were analyzed. To measure autophagy, cells were stained with acridine orange (AO) to detect vesicular organelles characteristic of lysosomal activity. Briefly, cells were cultured in medium, treated as described above and AO was added for 15 minutes at final concentration of 5 μg/mL. Cells were analyzed and photographed by using a fluorescence microscope with a digital camera (Leica DC 300F) and by using Leica IM1000 software (Leica Mycrosystem Ltd., Wetzlar, Germany).

### Cell lysis, immunoblotting

To analyze protein expression, treated and untreated cells were washed twice in 1X phosphate-buffered saline (PBS) and lysed in lysis buffer (50 mM Tris–HCl [pH 7.5], 150 mM NaCl, 2 mM EDTA, 0,1 % SDS, 0,5 % sodium deossycolate, 1 % TritonX-100, 1 mM PMSF, 20 μg/mL aprotinin, 20 μg/mL leupeptin). Lysates were clarified by high-speed centrifugation (14.000 rpm, at 4 °C x 30 min), and 40–60 μg of whole cell lysates were fractionated by SDS-PAGE. Proteins were subsequently transferred onto nitrocellulose membrane, and their expression was quantified by densitometric analysis and normalized for the expression of β-actin.

### Fluorescence microscopy and DAPI staining

To analyze proteins under non-denaturant conditions, RMS cells were treated with drugs or left untreated (DMSO) and then spotted onto 12-well multitest slides (ICN Biomedicals, Inc., USA). Cells were fixed with 4 % paraformaldehyde, permeabilized with 0.2 % TritonX-100 and non-specific reactivity was blocked by incubating cells with 100 mM glycin followed by 10 % FCS-PBS. Primary antibodies were added for 1 h at 37 °C, followed by fluorophore-conjugated secondary antibody Alexa488. Slides were washed in PBS, counter-stained with DAPI (4',6-diamidino-2-phenylindole, 1:5000) and mounted with 1:1 PBS-glycerol. Images were taken with a fluorescence microscope equipped with a digital camera (Leica DC 300F) and analyzed by using Leica IM1000 software (Leica Mycrosystem Ltd., Germany).

### Data analysis

Results were expressed as mean ± standard deviation (SD) of three independent experiments and analyzed by using the two-sided Student’s *t* test, with a P value < 0.05 considered significant.

## Results

### Combinatorial exposure of RMS cells to Bortezomib and 17-DMAG

**Figure 1:**
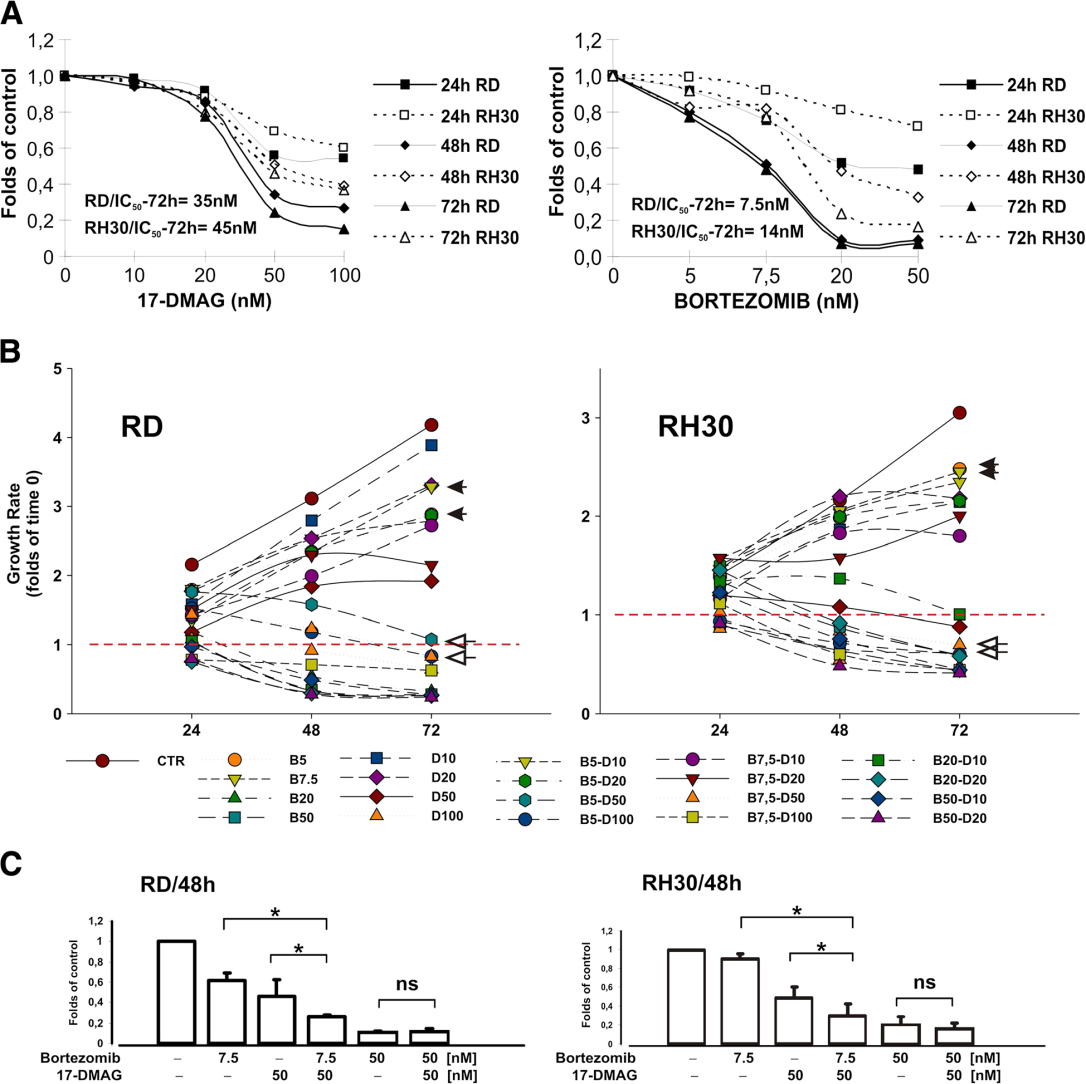
**Sensitivity of RMS cells to Bortezomib and/or 17-DMAG exposure.** (**A**) To assess cytotoxicity induced by Bortezomib and 17-DMAG, ARMS (RH30) and ERMS (RD) cells were exposed to increasing drug concentrations, and viability was measured by MTT assay after 24, 48, and 72 hours. Values, expressed as folds of control (untreated cells), represent means of three independent experiments. (**B**) Time- and dose-dependent effects of Bortezomib and 17-DMAG treatments were assessed in cells exposed to the thereof combination for up to 72 hours, using multiple drug concentrations (Bort. 5–7.5-20-50nM; 17-DMAG 10-20-50-100 nM) as indicated (*B*, Bortezomib; *D*, 17-DMAG). Cell viability was assessed by MTT assay, and data were expressed as described above. Values are means of three independent experiments. (**C**) Dose–response analysis of RH30 and RD cells treated for 48 hours with 7.5nM Bortezomib, 50nM 17-DMAG or the thereof combination is shown. Drug effectiveness of the Bortezomib/17-DMAG combination results significantly higher in comparison to that observed after both single-agent treatments (*t*-test analysis *, p < 0,05). Values are expressed as folds of control (untreated cells) and are means ± standard deviation of three independent experiments. ns: non significant.

To assess the optimal conditions for combinatorial use of Bortezomib and 17-DMAG in rhabdomyosarcoma tumour cells, the effects of Bortezomib and 17-DMAG as single agents were first investigated in ERMS (RD) and ARMS (RH30) cell lines, and cell viability was measured at increasing time points. In these conditions, Bortezomib and 17-DMAG inhibited proliferation of both RH30 and RD cells in a time- and dose-dependent manner, although embryonal RD cells resulted more sensitive to treatments, with a reduction in cell survival of up to 80 % after 72 hours with either Bortezomib (50 nM IC50 = 7.5nM) or 17-DMAG (100 nM IC50 = 35 nM) (Figure 1A). Drug treatments were less effective in alveolar RH30 cells, as indicated by both the higher IC50 values (Bortezomib IC_50_ = 14nM; 17-DMAG IC_50_ = 45nM) and the survival rate observed. Therefore, cytotoxicity of the Bortezomib/17-DMAG combination was assessed by using multiple drug concentrations and time points. Under these conditions, Bortezomib and 17-DMAG added simultaneously increased drug-induced growth inhibition of both cell lines, and the effect correlated with time exposure (Figure 1B). Combinations of low doses of Bortezomib (5–7.5nM) with cytostatic concentrations of 17-DMAG (≤50nM) (*open arrowheads*) were more effective than each compound used alone (*closed arrowheads*), whereas at higher concentrations (Bortezomib 20-50nM; 17-DMAG 100nM) cytotoxicity was independent of treatment modality (Figure 1B and 1C). In this context, Bortezomib/17-DMAG combination treatment was able to overcome the lower sensitivity of RH30 ARMS cells to proteasome inhibition, resulting more effective at inhibiting cell growth and survival than single-agent treatment.

### Simultaneous induction of apoptosis and autophagy in RMS cells

**Figure 2:**
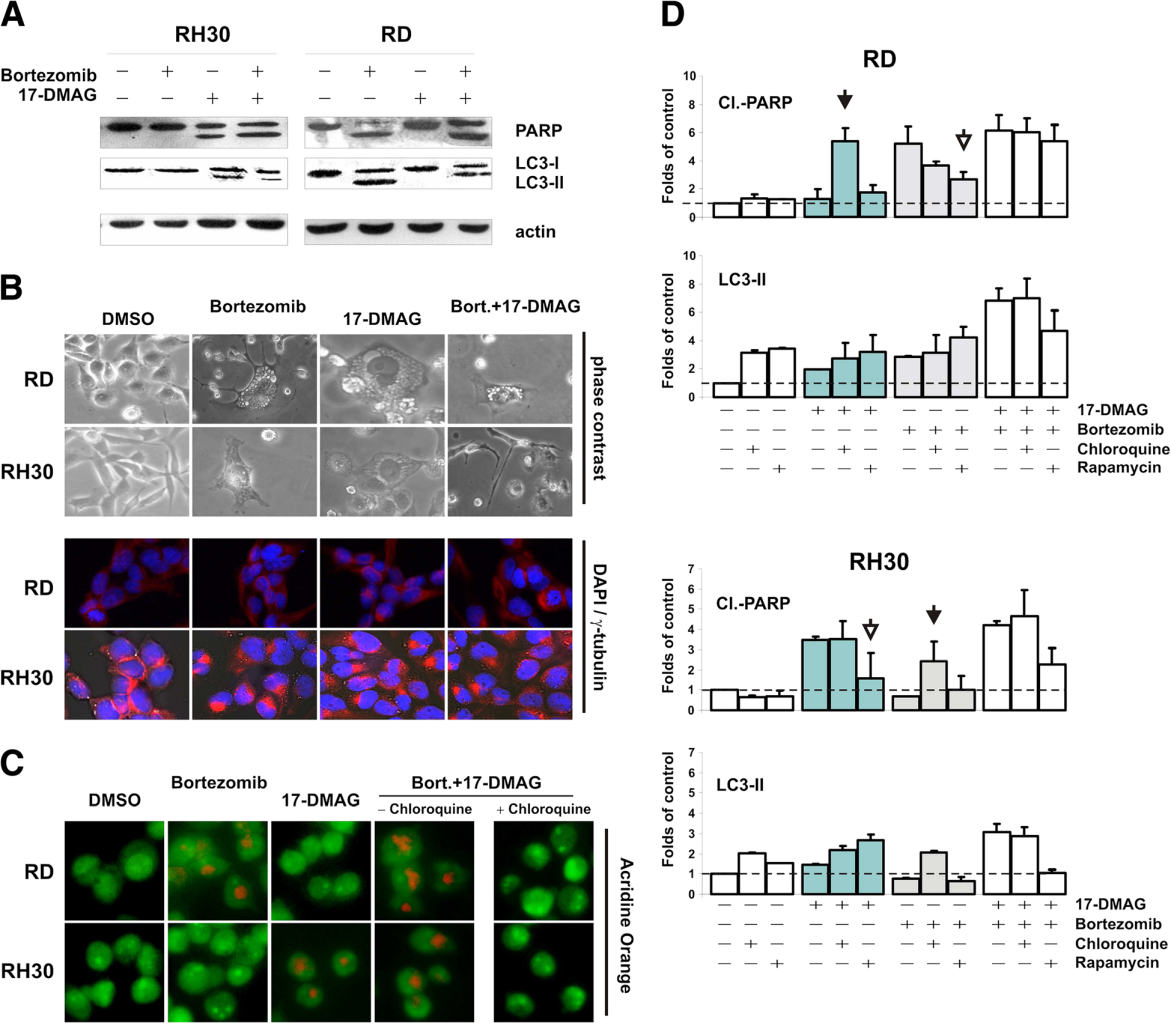
**Induction of autophagy in ARMS and ERMS cell lines.** (**A**) To assess apoptosis and autophagy induction, PARP and LC3 protein expression were determined by Western blot analysis, after 48 h-treatment with Bortezomib (7,5nM), 17-DMAG (50nM), or the combination Bortezomib/17-DMAG, using β-actin (actin) as loading control. (**B**) Under these conditions, analysis of RH30 and RD cell morphology and chromatin integrity was performed, using a contrast-phase microscope (63x magnification) and processing cells for DAPI/γ-tubulin staining. (**C**) Effects of single-agent or combinatorial treatments on vacuoles acidification were investigated by staining RH30 and RD cells with acridine orange (5 μg/mL), in the presence or absence of lysosomal inhibitor chloroquine. (**D**) Western blot analysis of cleaved PARP and processed LC3 (LC3-II) proteins in RH30 and RD cells treated with 48 hours with Bortezomib (7.5nM), 17- DMAG (50nM), or their combination, in the presence or absence of cloroquine or rapamycin. Proteins were analyzed by SDS-PAGE and band densities were measured with NIJ image software. Values are expressed as folds of control and are means ± standard deviation of three independent experiments.

Consistent with combination treatment results, 7.5nM Bortezomib and 50nM 17-DMAG were chosen as suitable concentrations to study the relationship between growth inhibition and survival, and to determine whether cross-talk between pathways influence RMS cell fate in the setting of proteotoxic stress. In this context, it was previously shown that proteasome inhibitors interfere with protein degradation and increase the amount of toxic aggregates in the cytoplasm [17-19], whereas heat shock protein inhibitors activate the unfolded protein response (UPR) pathway but also stimulate autophagy to maintain cell homeostasis [20-23]. Autophagy is an important mechanism for the removal of damaged cytoplasmatic components after cellular stress, but it can also lead to cell death in collaboration with several apoptogenic factors [24]. Therefore, to better understand the mechanisms that lead to growth inhibition and cell death of RMS cells in the presence of the Bortezomib/17-DMAG combination, we measured the levels of apoptosis and autophagy by monitoring both PARP cleavage and LC3 processing, respectively. When autophagy is up-regulated, cytosolic LC3-I protein is cleaved and recruited to autophagosome membranes (LC3-II) [25], where it drives autophagic vacuoles formation before lysosome-mediated proteolysis occurs [26]. Consistent with these findings, we observed that in these settings PARP cleavage occurred in RH30 exposed to 17-DMAG, whereas in ERMS RD cells apoptosis was induced by Bortezomib (Figure 2A). In both cell lines, the induction of apoptosis correlated with the conversion of endogenous LC3 protein from cytosolic LC3-I to the autophagosome-associated LC3-II form, and this occurred together with the accumulation of perinuclear vacuoles inside cells in the absence of a clear apoptotic morphology (Figure 2B, upper and lower panels). For further analysis of autophagy we used the lysosotropic compound acridine orange, measuring AO bright green-to-red fluorescence transition in acidic vesicles forming during autophagy [26]. Consistent with LC3-II accumulation, staining with acridine orange dye revealed an increase of red fluorescence in RD cells after treatment with Bortezomib, whereas red fluorescence in RH30 cells increased only after 17-DMAG exposure (Figure 2C). Treatment with both Bortezomib and 17-DMAG enhanced the amount of acidic vacuoles in either RH30 or RD cells, which was abrogated by the addition of chloroquine lysosomal inhibitor (Figure 2C). By interfering with luminal pH, chloroquine suppresses downstream lysosome-mediated autophagic degradation, whereas rapamycin, by stimulating both expression and processing of LC3- I, exerts an opposite effect and induces autophagy [27]. In addition, chloroquine in combination with proteasome inhibitors can enhance caspase-mediated apoptosis [28], whereas rapamycin when used in combination treatments reduces the accumulation of toxic poliubiquitinated aggregates and attenuates drug-induced cell death [29,30]. Therefore, the response pattern to Bortezomib and 17- DMAG of rhabdomyosarcoma cells was investigated in conditions of autophagy induction or repression, by adding to drug-treated and untreated cells rapamycin and chloroquine, respectively. We found that PARP cleavage increased in drug-treated RD and RH30 cells in the presence of chloroquine, but not after the addition of rapamycin (Figure 2D). In particular, suppression of autophagy by chloroquine correlated with the accumulation of cleaved PARP in cells not sensitive to either proteasome or Hsp90 inhibitors (i.e. RH30/Bortezomib and RD/17-DMAG, *closed arrowheads*), whereas stimulation of autophagy by rapamycin exerted the opposite effect primarily in cells that were sensitive to single agent treatments (RD/Bortezomib and RH30/17-DMAG, *open arrowheads*). In contrast, LC3-I processing in response to single agent treatments varied less significantly, as well as PARP cleavage in cells treated with the Bortezomib/17-DMAG combination (Figure 2D), suggesting that alteration of lysosomal function may be critical for cell survival at the early onset of stress response, but not following severe stress induction in late apoptosis.

### Autophagy as a pro-survival mechanism in apoptotic rhabdomyosarcoma cells

**Figure 3:**
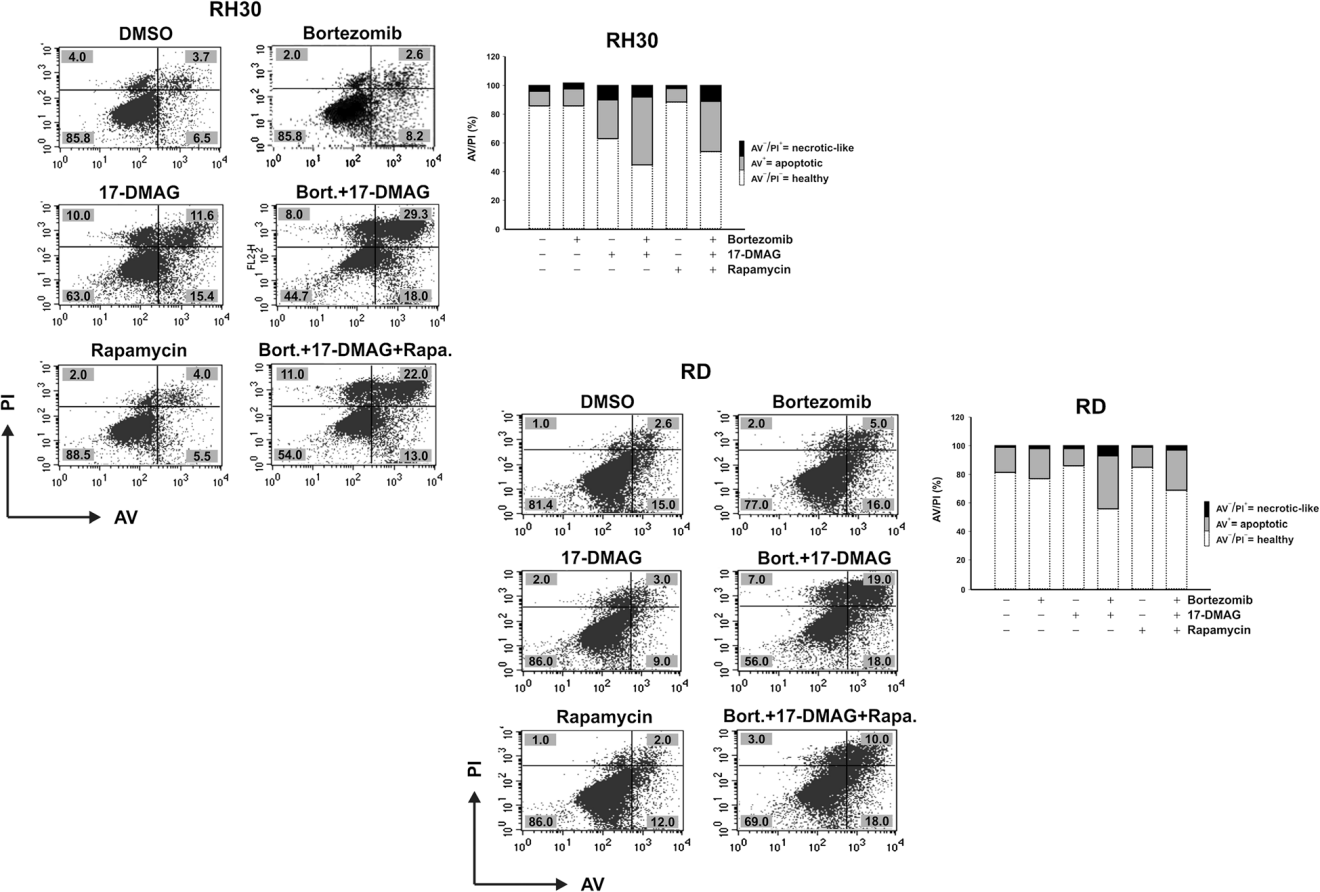
**Analysis of apoptosis induction in RH30 and RD Rhabdomyosarcoma cells.** To assess the role of autophagy in drug-treated RH30 and RD cells, apoptosis was measured by flow- cytometry after staining treated and untreated cells (Bortezomib 7,5nM; 17-DMAG 50nM; Bortezomib/17-DMAG) with Annexin-V-FITC/Propidium Iodide (AV/PI) in the presence or absence of non-toxic concentrations of rapamycin (5 ng/mL). Non-apoptotic (healthy, AV^−^/PI^−^), apoptotic (AV^+^/PI^−^ and AV^+^/PI^+^) and necrotic cells (AV^−^/PI^+^) were identified, and their relative percentages reported in the corresponding quadrants. The relative amount of each fraction is graphed aside.

The balance between apoptosis and autophagy is important in tumour development, but also for response to therapy, since in addition to their unique role in suppressing and promoting tumorigenesis, respectively, apoptosis and autophagy may contribute to drug sensitivity when interfacing to each other. To investigate further the involvement of autophagy in drug-induced apoptosis and cell survival, Annexin V/Propidium Iodide (AV/PI) double staining analysis was performed in rhabdomyosarcoma cells exposed to Bortezomib, 17-DMAG, or both, in the presence or absence of rapamycin. Consistent with previous findings, survival of RH30 and RD cells was significantly reduced by the Bortezomib/17-DMAG combination (Figure 3, *panels*, RH30/AV^−^/PI^−^ = 44.7 %; RD/AV^−^/PI^−^ = 56 %) compared with control and single-agent treatments. This was accompanied by increased apoptosis (Figure 3, *panels and histograms aside*, RH30/AV^+^ = 47.3 %; RD/AV^+^ = 37 %), whereas necrosis was not significantly induced (*panels*, RH30/PI^+^ = 8 %; RD/PI^+^ = 7 %, respectively). Importantly, pre-treatment with non-toxic concentrations of rapamycin partially suppressed apoptotic cell death caused by combined exposure to Bortezomib and 17-DMAG (Figure 3, *panels and histograms*), therefore supporting the notion that drug-induced autophagy may act as a compensatory mechanism in rhabdomyosarcoma tumour cells that mitigates stress response and contributes to apoptosis resistance.

## Discussion

The heat shock proteins (HSP) and the ubiquitin-proteasome system (UPS) contribute to maintain protein homeostasis in eukaryotic cells. They have been recently proposed as therapeutic targets in a wide variety of cancers [31,32]. Preclinical studies have demonstrated the antitumour activity of proteasome inhibitors in childhood sarcomas, including rhabdomyosarcoma, Ewing sarcoma (EWS) and osteosarcoma (OS) [7,33-36]. In addition, when used in combination with standard chemotherapeutic agents or targeted therapeutics, proteasome inhibitors can increase anti-tumor activity, suggesting that combination therapy can achieve better results than single-agent treatments [37,38]. Because several Hsp90 client proteins are relevant for tumour growth and survival, inhibition of Hsp90 has also been investigated in cancer, including rhabdomyosarcomas, demonstrating that targeting Hsp90 may reduce tumour cell proliferation and migration, both *in vivo* and *in vitro*[8,12]. Targeting the Hsp90 molecular chaperone has shown anti-tumor efficacy in Ewing sarcomas, whereas the efficacy in childhood osteosarcoma is lower and limited to specific treatment modalities [12,39,40]. Similarly to proteasome inhibitors, preclinical and clinical studies in haematological malignancies have demonstrated that Hsp90 inhibitors are effective as anti-cancer drugs especially when combined with conventional chemotherapy or targeted therapeutics [41-44]. Based on these observations, we investigated the antitumour activity of Bortezomib and the Hsp90 inhibitor 17-DMAG in rhabdomyosarcoma cells, comparing single-agent exposures with combination treatments. Consistent with previous findings [7,8], both Bortezomib and 17-DMAG were highly efficient at inhibiting growth and survival of RMS cells. Embryonal RD cells were more sensitive than alveolar RH30 cells to single-agent exposure, although such a different sensitivity was not justified by the expression of specific target proteins, like heat shock proteins, cell cycle inhibitors and pro-apoptotic factors [45,46]. Conversely, effectiveness of the combined treatment was comparable among cell lines, including RH30 cells that responded poorly to Bortezomib alone. Combination of Bortezomib and 17-DMAG induced autophagy in addition to apoptosis, and this was confirmed by the concurrent cleavage of LC3-II and PARP proteins.

Consistent with these findings, inhibition of proteasome activity causes the accumulation of misfolded proteins inside cells, which bind to heat shock proteins in discrete structures known as aggresomes and are subsequently degraded by lysosomes [47]. Attachment of ubiquitin to proteins, in fact, not only constitutes a degradation signal for the proteasome, but also serves for removal of proteins by lysosome-mediated autophagy [48]. In these conditions, unfolded proteins are delivered to lysosomes by heat shock proteins, in what is known as chaperone-mediated autophagy (CMA) [23,49-52]. Macroautophagy, the most important lysosome-mediated type of autophagy, does not usually need shuttling substrate proteins by heat shock proteins, but recent evidences suggest that in some cases heat shock proteins translocate ubiquitinated proteins into lysosomes and assist their autophagic degradation as well [48,53]. Collectively, these findings indicate that autophagy may be activated by proteasome inhibitors, likely as a response mechanism that alleviates from stress and protects cells from apoptosis [19,54-56]. Consistent with this scenario we demonstrated that autophagy is activated in rhabdomyosarcoma cells to withstand drug-induced cytotoxicity, as suggested by LC3-I activation and intensive cytoplasmic vacuolization. As expected, the inhibition of autophagy by chloroquine increases caspase-dependent PARP cleavage in rhabdomyosarcoma stressed cells, whereas its induction by rapamycin partially rescued cells from drug-induced apoptosis. In particular, inhibition of apoptosis occurs when cells are pretreated with rapamycin prior to administering Bortezomib and/or 17-DMAG, whereas the inhibition of autophagy increases cell death when induced together with both proteasome and Hsp90 inhibition. This suggests that RMS cells may activate autophagy as cytoprotective response to drug treatment, and the inhibition of autophagy enhances sensitivity of RMS cells to anti-cancer drugs, including Hsp90 and proteasome inhibitors. Of note, inhibition of autophagy is more effective at early onset of stress response than following apoptosis induction, providing evidence that autophagy occurs before cell death and it functions primarily as a cell survival mechanism.

The ubiquitin-proteasome and autophagy-lysosome are often considered distinct degradation systems. However, recent studies suggest that these two pathways are mechanistically linked [53,56], as proteasome inhibition induces autophagy when removal of toxic polyubiquitinated aggregates is necessary for cell survival, while proteasome activity is induced when formation and activity of lysosomes are impaired. Autophagy and apoptosis are events regulated by common survival pathways, including the JNK1, Bcl-2 and the PI3K/AKT signaling pathway. It has been shown that JNK-dependent phosphorylation of Bcl-2 promotes cell survival by disrupting Bcl-2 binding to Beclin-1 and activates autophagy, whereas sustained Bcl-2 phosphorylation blocks Bcl-2 anti-apoptotic activity and apoptosis overwhelms autophagy [57,58]. AKT phosphorylates and prevents Bad pro-apoptotic activity and inhibits autophagy by impairing TSC1TSC2 tumor suppressor proteins activity [59]. Of note, JNK and AKT are, among survival proteins, mostly affected in rhabdomyosarcoma cells treated with proteasome and Hsp90 inhibitors, but their involvement in drug-induced autophagy have not been investigated yet [7,8,60].

Nevertheless, our findings suggest that combination treatment with Bortezomib and 17-DMAG can overcome autophagy, a mechanism protecting rhabdomyosarcoma cells from drug-induced cytotoxicity. Further studies are warranted on the use of low concentrations of proteasome inhibitors in combination with Hsp90 inhibitors, both *in vitro* and *in vivo,* as they might represent a tool capable of counteracting protective mechanisms, such as autophagy, that may affect treatment efficacy and, ultimately, the outcome of RMS patients.

## Conclusions

Our study showed that the combination of Bortezomib with 17-DMAG exerts more potent inhibitory effects on RMS cell growth than each agent alone. Combination treatment has important therapeutic advantages because it counteracts survival mechanisms that occur as side effects of treatment. These results may contribute to new therapeutic approaches in rhabdomyosarcoma.

## Abbreviations

17-DMAG, [17-(Dimethylaminoethylamino)-17-Demethoxygeldanamycin]; AO, Acridine Orange; ARMS, Alveolar Rhabdomyosarcoma; AV, Annexin V-FITC; BZ, Bortezomib; CMA, Chaperone Mediated Autophagy; ERMS, Embrional Rhabdomyosarcoma; Hsp90, Heat Shock Protein 90; HSR, Heat Shock Response; LC3, Light Chain 3; MTT, 3-(4,5-dimethylthiazol-2-yl)-2,5-diphenyltetrazolium bromide; PI, Propidium Iodide; RMS, Rhabdomyosarcoma; UPR, Unfolded Protein Response; UPS, Ubiquitin-Proteasome System.

## Competing interests

The authors declare that they have no competing interests.

## Authors’ contributions

MP and PB designed and conducted the study and analyzed the data. AR designed the study and coordinated all aspects of the project. All authors contributed to write, read and approve the manuscript in the final form.

## Pre-publication history

The pre-publication history for this paper can be accessed here:

http://www.biomedcentral.com/1471-2407/12/233/prepub
